# Soft-Shelled Turtle Peptides Extend Lifespan and Healthspan in *Drosophila*

**DOI:** 10.3390/nu14245205

**Published:** 2022-12-07

**Authors:** Qianqian Wang, Junhui Zhang, Jiachen Zhuang, Fei Shen, Minjie Zhao, Juan Du, Peng Yu, Hao Zhong, Fengqin Feng

**Affiliations:** 1 College of Biosystems Engineering and Food Science, Zhejiang University, Hangzhou 310058, China; 2 Zhejiang Nuoyan Biotechnology Co., Ltd., Huzhou 313000, China; 3 Yuyao Lengjiang Turtle Industry, Ningbo 315400, China; 4 College of Food Science and Technology, Zhejiang University of Technology, Hangzhou 310014, China

**Keywords:** soft-shelled turtle peptide, lifespan, healthspan, TOR, molecular docking

## Abstract

In traditional Chinese medicine, soft-shelled turtle protein and peptides serve as a nutraceutical for prolonging the lifespan. However, their effects on anti-aging have not been clarified scientifically in vivo. This study aimed to determine whether soft-shelled turtle peptides (STP) could promote the lifespan and healthspan in *Drosophila melanogaster* and the underlying molecular mechanisms. Herein, STP supplementation prolonged the mean lifespan by 20.23% and 9.04% in males and females, respectively, delaying the aging accompanied by climbing ability decline, enhanced gut barrier integrity, and improved anti-oxidation, starvation, and heat stress abilities, while it did not change the daily food intake. Mechanistically, STP enhanced autophagy and decreased oxidative stress by downregulating the target of rapamycin (TOR) signaling pathway. In addition, 95.18% of peptides from the identified sequences in STP could exert potential inhibitory effects on TOR through hydrogen bonds, van der Walls, hydrophobic interactions, and electrostatic interactions. The current study could provide a theoretical basis for the full exploitation of soft-shelled turtle aging prevention.

## 1. Introduction

Aging is defined as biological and physiological processes at the late phase of life, typically characterized by the increased functional decline of organs and subsequent organismal death. The onset of age-related disorders such as atherosclerosis, type 2 diabetes, osteoporosis, neurodegeneration, and cardiovascular disease is frequently accompanied by the decay process [1,2]. Thus, strategies delaying the aging process should increase both lifespan and healthspan because most people are not only concerned about how long they will live but also care more about the length of time they can have good health and the ability to deal with daily activities [3,4].

Bioactive peptides, which are produced by proteolysis from food protein, have been proven to prolong lifespan and suppress the incidences of age-linked illnesses like cancer, cognitive decline, and neurodegeneration [5,6,7,8,9]. Soft-shelled turtles (*Pelodiscus sinensis*) have been widely consumed as a food and medicine with high nutritional value since ancient times. Especially, the traditional remedy of soft-shelled turtles in oriental medicine claims a longevity-promoting effect. A previous study demonstrated that soft-shelled turtle peptide (STP) supplementation could reduce physical exhaustion and oxidative stress as well as enhance exercise endurance and energy metabolism by modulating the oxidative stress-related protein in mice [10]. Due to the relationship between oxidative stress and aging, it is necessary to explore the anti-aging effect of STP. However, few reports explored the longevity-promoting and direct anti-aging effects of STP [11]. Additionally, previous studies primarily concentrated on confirming the function effect of STP, whereas the structural properties of peptides and structure-activity relationship remain unknown [10,12]. Hence, it is essential to identify and classify effective anti-aging peptides from STP.

Previous studies reported that the target of the rapamycin (TOR) signaling pathway was necessary for the lifespan promotion effect of some bioactive peptides [13,14]. TOR, a highly conserved serine/threonine kinase of the phosphatidylinositol kinase-related kinase family, is essential for sensing and responding to nutrient stimuli as well as delivering growth signaling in eukaryotes [15]. TOR complex 1 (TORC1) and TOR complex 2 (TORC2) are the two function complexes for TOR. Multiple TORC1-regulated processes appear to coordinately and overlappingly contribute to the pro-longevity effects of TORC1 inhibition [16]. Studies indicated that TOR signaling is a ROS-sensitive pathway, and the antioxidant effects of substances could affect the expression of TOR [17,18]. Regarding the in vivo antioxidant activity of STP, the idea that the exploration of STP to regulate the expression of TOR to increase autophagy and inhibit oxidative stress for longevity-promoting was practicable.

Here, we utilized *Drosophila* as a model organism to investigate the lifespan and healthspan extension effects of STP, and further identify metabolic pathways that correlate with lifespan. Simultaneously, the possible mechanism underlying the anti-aging effect of STP were elucidated by measuring the gene expression levels of the TOR signaling pathway. Moreover, the peptide profiles of STP were investigated as possible anti-aging peptides by molecular docking.

## 2. Materials and Methods

### 2.1. Materials

Wild-type Canton-S strain of *Drosophila melanogaster* was obtained from the Institute of Food Bioscience and Technology of Zhejiang University. The obtained flies were kept under controlled conditions at a temperature of 25 °C, humidity of 65%, and a 12-h light–dark cycle. Soft-shelled turtle peptides were kindly provided by Hangzhou Kangyuan Food Science and Technology Co., Ltd. (Hangzhou, China). The basic chemical components, relative molecular mass distribution, and amino acid composition of STP were shown in our previous study [10].

### 2.2. Fly Husbandry and Treatment

The parental flies were inoculated at a ratio of 20 males: 40 females and were cultured in the basal medium. Then, the parental flies were released on the fifth day. Afterward, the offspring of flies were reared at a standard larval density of ~300 flies per bottle, and all enclosed adults were collected over a 12-h period to obtain the F1 generation. We repeated this procedure to produce the F2 generation of flies. After the eclosion of the embryos of the F2 generation, male and female flies were collected within 48 h under CO_2_ anesthesia for further experiments. We refer to the first day of a dietary treatment as the first day of adulthood for flies. The flies were reared on basal medium or soft-shelled turtle peptide-supplemented medium. The basal medium contained corn meal (10.5%, *w*/*v*), yeast (4%, *w*/*v*), sucrose (7.5%, *w*/*v*), agar (0.75%, *w*/*v*), and propionic acid (1%, *v*/*v*) (control group, CT). Soft-shelled turtle peptide-supplemented medium was prepared by adding soft-shelled turtle peptide power into a cooled (65 °C) liquid basal medium with a concentration of 0.8% (*w*/*v*) STP.

### 2.3. Lifespan Analysis

Flies (including male and female) were harvested within 48 h after hatching and anesthetized by light CO_2_ for sortation, after which the flies were transferred to 30-mL vials containing treatment medium (CT or STP) with a density of 30–35 flies per vial. A total of 10 vials were set up for the CT and STP groups. After every 3–4 days, these flies were again transferred to new vials containing several types of media. All the dead flies were counted until no survivors remained. The mean, median, and maximum lifespan were calculated in accordance with the previously reported method [13].

### 2.4. Feeding Assay

Flies in the CT and STP groups were kept for starvation in (1%) agar for 24 h on day 30. Afterward, flies were cultured in the darkness for 4 h on agar containing F&D blue No. 1 (0.5%, Shanghai Macklin Biochemical Co., Ltd., Shanghai, China). Then, the flies were frozen on liquid nitrogen immediately and measured [19].

### 2.5. Climbing Assay

The evaluation of climbing ability was conducted as previously described [20]. On the 40th day, empty tubes with a line 6 cm above the bottom were used to keep both STP and CT groups of flies. After transferring the flies into containers, flies were gently trapped at the bottom; a ten-second timeline was recorded for flies to cross the drawn line.

### 2.6. Smurf Assay

The intestinal barrier function was evaluated using the smurf assay, as published previously [21]. On the 40th day, flies were transferred into new vials with the medium containing F&D blue No. 1 (2.5%) for 9 h. Flies with gut stains were counted as a smurf.

### 2.7. Stress Assay

Flies were fed for 30 days before a stress assay. A total of 6 vials (180–210 flies) were set up for CT and STP groups. For the oxidative stress assay, flies were fed H_2_O_2_ (30%) dissolved in glucose (5%) supplied on filter paper [13]. In the starvation test, flies were placed in tubes with agar (1%) [22]. The survival rate was documented every five hours. For the heat stress assay, flies were placed in an empty tube at 37 °C, and the survival rate was documented every half hour [23]. For the cold stress assay, flies were placed in empty vials bathed on ice at 4 °C for 2 h, and then placed at a temperature of 25 °C for behavior recording for 2 h [24].

### 2.8. Determination of Biochemical Index

Flies aged 30 days were immersed in liquid nitrogen and then kept at −80 °C for further examination. Commercial kits from the Nanjing Jiancheng Bioengineering Institute, Nanjing, China, with the manuals were used to determine the protein content (Catalog No. A045-3-2), malondialdehyde (MDA, Catalog No. A003-1-2), triacyleglyceride (TAG, Catalog No. A110-1-1), glutathione peroxidase (GSH-PX, Catalog No. A005-1-2), and superoxide dismutase (T-SOD, Catalog No. A001-1-2).

### 2.9. Untargeted Metabolomics Analysis

The whole flies at 30 days of age were collected, and the sample preparation methods of metabolomics analysis were assessed as reported previously [25]. Briefly, samples were separated using Agilent 1290 UHPLC (Agilent Technologies, Santa Clara, CA, USA) equipped with the ACQUITY UPLC BEH Amide column (1.7 μm, 2.1 mm × 100 mm), and analyzed by Triple 6600 TOF mass spectrometer (AB Sciex, Concord, Toronto, ON, Canada). Metabolites were identified based on the exact mass of their MS and tandem MS spectra, which were then searched and compared using a laboratory database (Shanghai Applied Protein Technology Co., Ltd., Shanghai, China). The initially processed data were enumerated with SIMCA software (V14.1, Umetrics, Ume, Västerbotten, Sweden) for mode identification following normalization to total peak intensity (by weight of the complete flies). Following the collection of valid data, principal component analysis (PCA) and orthogonal partial least-squares discriminant analysis (OPLS-DA) were applied to differentiate STP from the CT group. Variable importance in projection (VIP) > 1 and *p* < 0.05 were employed as criteria for screening potential biomarkers, and the KEGG metabolomics pathway analysis was constructed to reveal the most relevant pathway for STP to exert anti-aging effects.

### 2.10. Real-Time Quantitative PCR

The detailed protocol of RT-qPCR was performed as reported previously in our laboratory [10]. Primers were provided by Tsingke Biotechnology Co., Ltd. (Beijing, China). RP49 was used as a reference by using the 2^(−ΔΔCt)^ method, and the designed primer sequences are shown in Table S1.

### 2.11. Peptides Sequence Identification

STP was desalted using a C18 stage tip before lyophilization according to the method of Hu et al. [26] and analyzed by EASY-nLC-orbitrap MS/MS system. The detailed information was shown in our previous study [27].

### 2.12. Molecular Docking of STP on FKBP12-FRB

The interaction between the FKBP12-FRB (as the receptor, PDB ID: 3FAP) and peptide identified from Section 2.11 (as the ligands) was conducted by molecular docking (Discovery Studio software, Accelrys, San Diego, CA, USA) to predict the potential inhibitory activity of peptides with anti-aging activity [15]. The structure of FKBP12-FRB was optimized via operations cleaning, preparation, dehydration, and hydrogenation operation. The docking program was conducted with special binding sites (coordinates: x = 10.8388, y = 24.8616, z =36.6092) and a set receptor radius (25.00 Å).

### 2.13. Statistical Analyses

The statistical analysis was accomplished with GraphPad prism 6.0. All values signify means ± SEM, and *p*-value ˂ 0.05 was taken as statistically significant. The Two-tailed unpaired *t*-test was used to analyze the comparisons between two independent groups.

## 3. Results

### 3.1. STP Extended Lifespan and Healthspan in Drosophila

**Figure 1:**
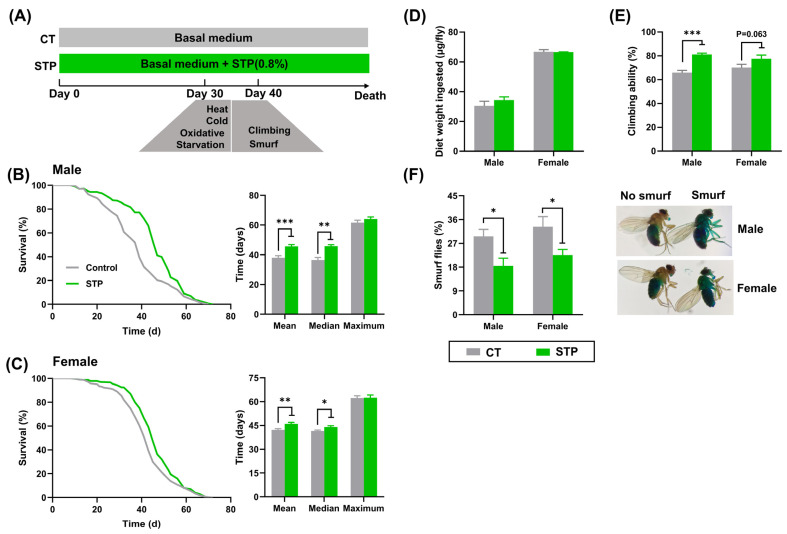
STP extended lifespan and improved healthspan in flies. (**A**) The scheme for animal experiment; Lifespan curves of male flies (**B**) and female flies (**C**); (**D**) Food intake; (**E**) Climbing ability; (**F**) Smurf flies. Data are shown as mean ± SEM. Statistical test: two-tailed unpaired *t*-test (* *p* < 0.05, ** *p* < 0.01, and *** *p* < 0.001 vs. CT group).

As shown in Figure 1B,C, the survival curves of flies in the STP group were right-skewed compared to the CT group, with the increase of mean, median, and maximum lifespan by 20.23% (*p* < 0.001), 25.48% (*p* < 0.01), and 4.03% in male flies, 9.04% (*p* < 0.01), 6.02% (*p* < 0.05), and 0.31% in females, respectively. Flies in the STP group exhibited slightly increased average food consumption than the CT group, but this was not statistically significant (Figure 1D). Furthermore, STP supplementation increased the climbing ability by 23.19% (*p* < 0.001) for male flies and 10.55% for females (*p* = 0.063) compared to the CT group (Figure 1E). Similarly, STP decreased the number of smurf flies by 37.74% (*p* < 0.05) for male flies and 32.09% (*p* < 0.05) for females compared to the CT group (Figure 1F). Taken together, these results suggested that STP extended the lifespan and healthspan in both male and female flies without limiting food intake. Moreover, the male flies that received STP exhibited better performance than females. Hereafter, our experiments focused on male flies.

### 3.2. STP Improved Stress Resistance in Drosophila

**Figure 2:**
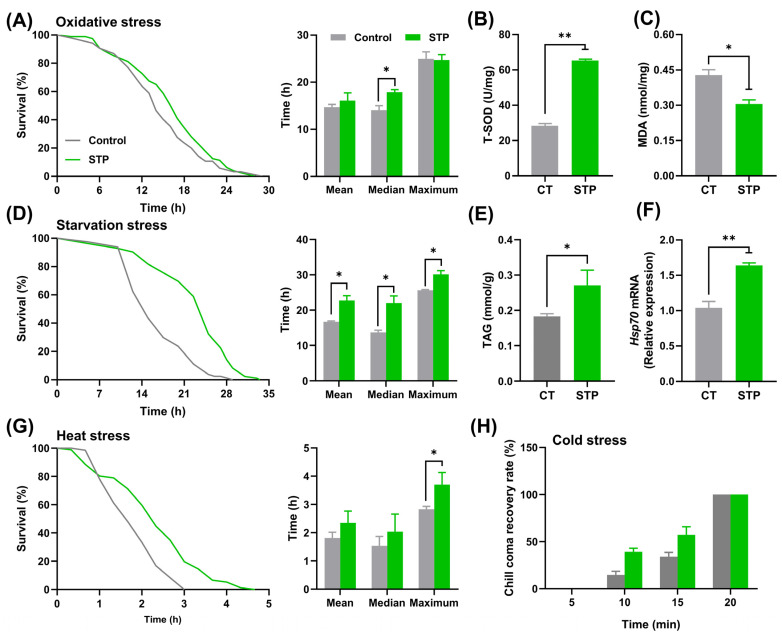
STP improved the resistance ability to stress in male flies. (**A**) Oxidative stress; (**B**) T-SOD activity; (**C**) MDA content; (**D**) Starvation stress; (**E**) TAG content; (**F**) Gene expression level of *Hsp70*; (**G**) Heat stress; (**H**) Cold stress. Data are shown as mean ± SEM. Statistical test: two-tailed unpaired *t*-test (* *p* < 0.05 and ** *p* < 0.01 vs. CT group).

STP enhanced the survival rate of male flies under oxidative stress, and the mean, median, and maximum lifespan were increased by 9.44%, 27.38% (*p* < 0.05), and −0.89%, respectively, compared to the CT group (Figure 2A). The T-SOD activity increased (*p* < 0.01), whereas the MDA content decreased (*p* < 0.05) (Figure 2B,C). Moreover, STP increased the survival rate of male flies under starvation stress with the extension of mean, median, and maximum lifespan by 35.97% (*p* < 0.05), 60.98% (*p* < 0.05), and 17.79% (*p* < 0.05), respectively (Figure 2D). Accordingly, the TAG level was increased via STP supplementation by 48.26% (*p* < 0.05) (Figure 2E). In addition, STP enhanced the survival rate of male flies under heat stress, which corresponds with the upregulation of *Hsp70* mRNA expression in comparison with the CT group (*p* < 0.01) (Figure 2F). The mean, median, and maximum lifespan in STP under heat stress were prolonged by 29.44%, 32.61%, and 30.72% (*p* < 0.05), respectively (Figure 2G). However, STP supplementation has no significant impact on the resistance capability under cold stress conditions (Figure 2H). These findings implied that STP could improve the stress resistance of flies to oxidation, heat, and starvation.

### 3.3. STP Impacted the Potential Metabolic Markers in Pathways Associated with Aging in Drosophila

**Figure 3:**
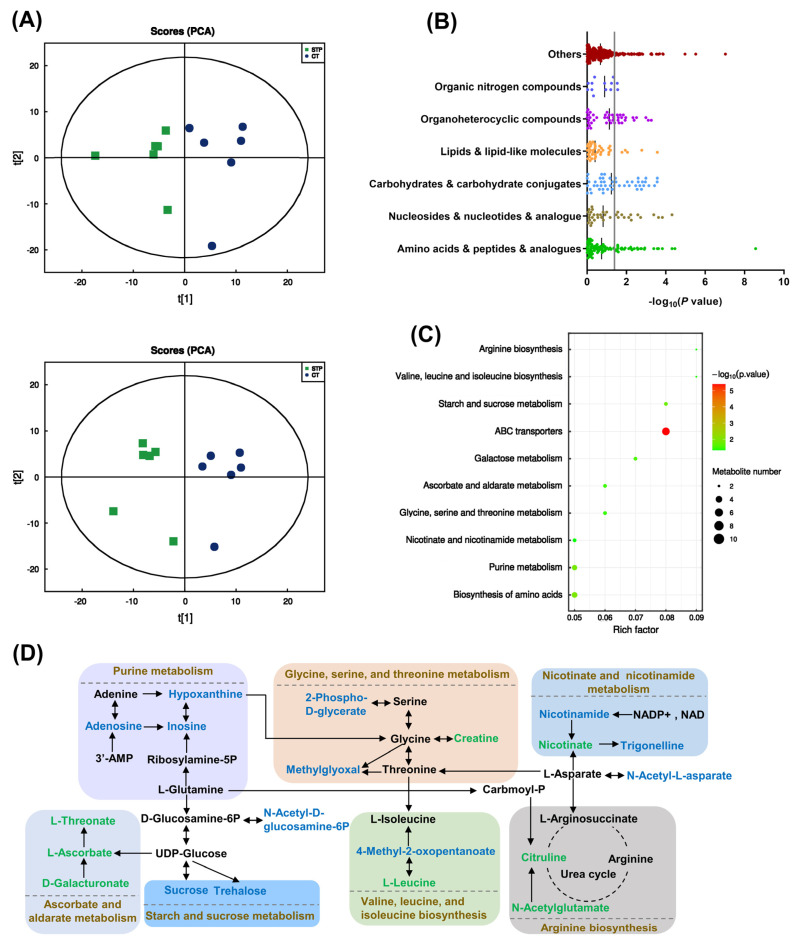
STP modulated metabolome in male flies. (**A**) The PCA scores plot under positive and negative ion modes; (**B**) Significant altered metabolites between CT and STP groups; (**C**) KEGG pathway resulting from the differential metabolites; (**D**) Metabolic pathways affected by STP supplementation in male flies. Metabolites colored in green/blue represent metabolites with increased/decreased levels after STP supplementation.

A clear separation of metabolites between the two groups was shown in the PCA plot (Figure 3A), indicating that STP supplementation could induce significant metabolic changes in flies. As shown in Figure 3B, 74 significant differential metabolites were identified mainly including amino acids, peptides, and analogs (8), nucleosides (8), carbohydrates and carbohydrate conjugates (13), lipids and lipid-like molecules (3), organoheterocyclic compounds (12), and organic nitrogen compounds (2). The KEGG pathway enrichment study revealed that STP intervention significantly affected 10 metabolic pathways (Figure 3C). For instance, compared to the CT group, metabolites (hypoxanthine, adenosine, and inosine) closely related to the purine metabolism pathway were downregulated after STP treatment. Nicotinamide and trigonelline, which are involved in the pathway of nicotinate and nicotinamide metabolism, were downregulated, whereas the nicotinate was upregulated. Moreover, compared to the CT group, 2-phospho-D-glycerate, methylglyoxal, and 4-methyl-2-oxopentanoate, crucial to the amino acid metabolism and biosynthesis (including glycine, serine, and threonine metabolism, arginine biosynthesis, valine, leucine, and isoleucine biosynthesis), were found downregulated after STP treatment, while creatine, citrulline, N-acetylglutamate, and L-leucine upregulated. Raffinose, D-galactonate, sucrose, and trehalose were downregulated, which are involved in the carbohydrate metabolism (ascorbate and aldarate, starch and sucrose, and galactose metabolism), and L-ascorbate, L-threonate, and D-galacturonate were upregulated. The main pathways affected by STP were summarized and sketched in Figure 3D.

### 3.4. STP Regulated TOR Signaling-Related Genes in Drosophila

**Figure 4:**
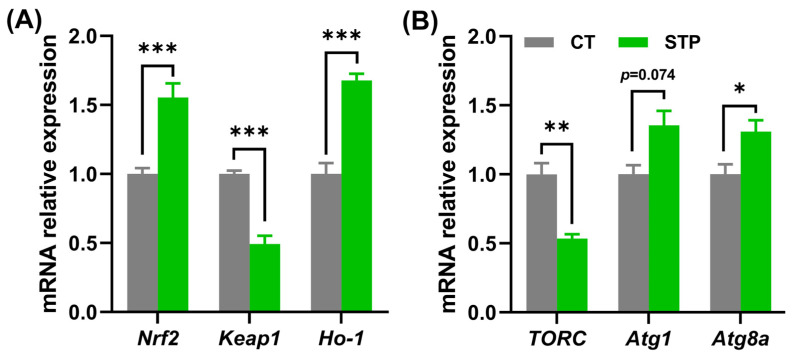
STP may inhibit the TOR signaling pathway to prolong the lifespan of flies. The gene expression level of (**A**) *Nrf2*, *Keap1*, and *Ho-1*; (**B**) *TORC*, *Atg1*, and *Atg8a*. Data are shown as mean ± SEM. Statistical test: two-tailed unpaired *t*-test (* *p* < 0.05, ** *p* < 0.01, and *** *p* < 0.001 vs. CT group).

As presented in Figure 4A, compared to the CT group, STP has substantially significantly upregulated the relative mRNA expression of nuclear factor-erythroid-2-like 2 (*Nrf2*) and heme oxygenase-1 (*Ho-1*) (*p* < 0.001), whereas downregulated kelch-like ECH-associated protein 1 (*Keap1*) (*p* < 0.05) in flies. Also, STP significantly downregulated the relative mRNA expression of *TORC* and upregulated the expression of autophagy-related gene1 (*Atg1*) (*p* = 0.0714) and autophagy-related gene 8a (*Atg8a*) (*p* < 0.05) in male flies compared to the CT group (Figure 4B). Taken together, the current data suggested that STP could enhance autophagy and inhibit oxidative stress by inhibiting TOR signaling pathway in flies.

### 3.5. Prediction of Peptides from STP with Anti-Aging Activity by Molecular Docking

**Figure 5:**
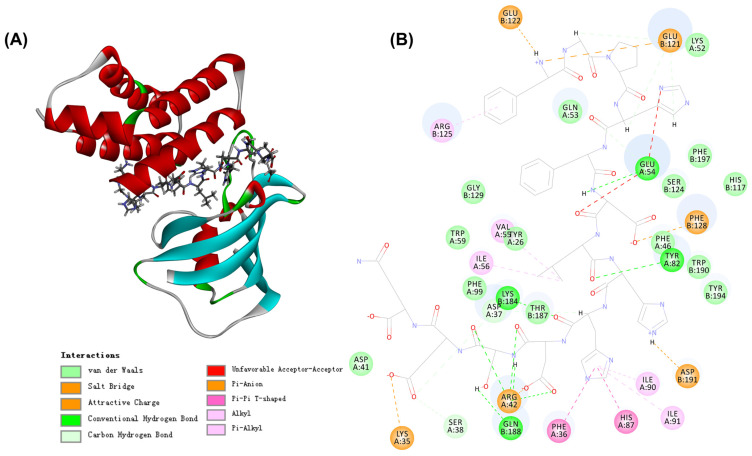
The 3D (**A**) and 2D (**B**) diagrams of the interactions between ADLETYLLEKSRVT and FKBP12-FRB.

In total, 187 peptide sequences were identified from STP. Table S2 revealed that the molecular weight of peptide sequences ranged between 330.1903 and 1901.9041 Da in STP, and the sequences were majorly constituted by 4–16 amino acid residues. Next, the molecular docking of these sequences with FKBP12-FRB was performed to predict potential peptides with anti-aging activity. A total of 178 peptides, accounting for 95.18% of CPTP, were successfully docked (Table S2). Among them, the #91 peptide sequence ADLETYLLEKSRVT displayed the highest (-) CDOCKER Energy value of 225.071, indicating that this peptide may have the highest docking potential for the target. Moreover, the molecular interaction between ADLETYLLEKSRVT and FKBP12-FRB is shown in Figure 5. Four weak interactions, i.e., hydrogen bonds, van der Waals, hydrophobic, and electrostatic interactions, were observed from their simulated docking with FKBP12-FRB. To be more specific, ADLETYLLEKSRVT comprising 8 residues, had 15 hydrogen bonds with Ser38, Asp37, Arg42, Tyr42, and Glu54 on the A chain and Lys184, Gln188, and Glu121 on the B chain. There were 14 van der Walls with Asp41, Phe99, Trp59, Tyr26, Phe46, Gln53, and Lys52 on the A chain and Thr187, Gly129, Tyr194, Trp190, His117, Ser124, and Phe197 on the B chain. As for the hydrophobic and electrostatic interactions, ADLETYLLEKSRVT had seven hydrophobic interactions with Phe36, His87, Ile91, Ile90, Ile56, and Val55 on the A chain, Arg125 on the B chain, while there were six electrostatic interactions with Lys35 and Arg42 on the A chain, and Asp191, Phe128, Glu121, and Glu122 on the B chain. In short, hydrogen bonds, van der Waals, and hydrophobic and electrostatic interactions may help the peptide form a more stable complex with FKBP12-FRB.

## 4. Discussion

Bioactive peptides like cultured crocodile meat hydrolysates, sea cucumber hydrolysate, and crimson snapper peptides could increase the survival curve (indicative of increased lifespan) [5,28,29]. Similarly, STP supplementation prolonged the mean and median lifespan in both male and female flies, suggesting that STP was effective in the anti-aging of flies. Nevertheless, aging not only increases the chance of mortality, but also reduces physiological, physical, cognitive, and reproductive functions [30]. In the study, the age-related changes in muscle/neuronal function (climbing ability) and intestinal function (smurf flies) were improved by STP supplementation, which suggested that STP-treated flies were in better health than the CT group at the same age. It is reported that the incidence of intestinal dysfunction increases with age, which leads to an increased intestinal microbial load and a reduction in the host lifespan [31]. The lifespan of flies with intestinal barrier dysfunction increased with the removal of the microbes [31,32]. Therefore, STP may have reduced the microbial load to maintain the intestinal barrier function in flies, thus prolonging the lifespan of flies. Notably, the current data showed that the effect of STP on the lifespan and healthspan was more obvious in male flies than females, which suggested that the beneficial effects of STP are related to sex. Likely, Chen et al. found that crimson snapper peptides could significantly increase the healthspan in male flies but not in females [29]. This difference may be due to the higher energy required by female flies to reproduce as the possible occurrence of mating behavior. Intriguingly, it has been reported that such differences in responses to bioactive peptides between gender also exists in rodents subjected to caloric restriction [33]. One hypothesis to crack this puzzle is that the gender-biased physiologic utilization and allocation of energy difference will affect the response to daily energy alterations in males and females [34]. However, further studies on this sexual difference are greatly needed.

Generally, the phenotype alterations of longevity are accompanied by the altered resistance ability to various environmental stresses in flies. Since STP increased the lifespan and healthspan of flies, we subsequently tested whether it would enhance resistance to stress and thus lead to an increased survival rate in flies. As expected, STP supplementation could increase the survival rate in flies under oxidative stress. Correspondently, the increased antioxidant enzyme activity and decreased MDA content in the STP group further corroborated that STP could enhance the resistance to oxidative stress in flies, which was also observed in mice [10]. Similar results were found in starvation stress, and significant increases in TAG content further confirmed the finding. TAG, one of the most crucial lipid-storage molecules in insects, has been reported to affect the survival rate under starvation conditions to a great extent [35]. Additionally, STP increased the resistance to heat stress and was accompanied by significantly increased *Hsp70* gene expression, which was in agreement with the investigations of Su et al. [36]. Nevertheless, STP supplementation failed to increase the resistance to cold stress, which might be caused by the short time in the ice bath (flies in the CT and STP groups were woken up within twenty minutes). Taken together, these findings imply that STP could improve the stress resistance of flies to oxidation, heat, and starvation.

The mechanism that regulates the lifespan of an organism involves numerous metabolic changes. For a better understanding of the impact of STP on the metabolite composition, the untargeted metabolomics technique was further utilized to analyze the metabolic profiles of the whole flies in the CT and STP groups. In flies, purine metabolites such as adenosine and inosine were upregulated when the lifespan was shortened by dietary high-purine, high-sugar, or high-yeast [37] but decreased after STP treatment. Purine metabolism was used to maintain an optimal level of nucleotides in the tissues, which play a crucial role in the biochemical processes of energy metabolism. Additionally, the levels of creatine, citrulline, and L-ascorbate were upregulated after STP treatment. Aon et al. discovered that glycine–serine–threonine metabolism was one of the primary pathways of mouse longevity [38]. Supplementation with creatine, which is involved in glycine–serine–threonine metabolism, can reduce brain energy expenditure, oxidative stress, and mitochondrial dysfunction, thus ultimately improving cognitive performance in the elderly [39]. Citrulline, a component of arginine biosynthesis, has a powerful antioxidant capability to enhance long-term potentiation in aged rats [40]. L-ascorbate could ameliorate brain aging through antioxidative and anti-inflammatory effects [41]. Metabolomics findings suggested that the anti-aging activity may be linked to inhibited oxidative stress.

In the study, the molecular weight of <1000 Da of STP was 93.23%. It is reported that small peptides could interact with free radicals more efficiently through their ability to touch the intestinal barrier easily in vivo [42]. Additionally, seventeen amino acids were found in STP with a total content of 761.17 mg per g. Tyr, Cys, Met, His, Asp, Glu, Ala, Val, Pro, Phe, and Leu may contribute to the antioxidant activity of peptides [43]. In the current study, the content of the above antioxidative amino acids was 462.14 mg per g (accounting for 60.71% of STP). The results were consistent with the increased resistance to oxidative stress. Interestingly, the tolerance to oxidative stress and lifespan in flies = regulated the Nrf2/Keap1 signaling pathway [44]. Under normal conditions, Nrf2 (a stress-responsive transcription factor) is sequestered in the cytosol by Keap1 to maintain Nrf2 in an inactive state. However, the binding state is disrupted when exposed to oxidative stress, and then Nrf2 translocates to the nucleus to regulate the expression of more than a hundred genes [45]. Compared to the CT group, STP treatment significantly upregulated and downregulated the *Nrf2* and *Keap1* gene expression levels. Also, the downstream target gene (*Ho-1* and *Gclc*) expression was markedly upregulated after STP treatment. Those results implied that STP regulated the Nrf2/Keap1 signaling pathway to inhibit oxidative stress, which was consistent with the findings of increased tolerance to oxidative stress and metabolomics. Wu et al. reviewed that the homeostasis of intestinal microbiota and the balance of reactive oxygen species in the gut can be altered by bioactive peptides [46]. Therefore, STP may play a critical role in maintaining gut health and function by inhibiting oxidative stress. It is interesting to note that studies have shown that the inhibition of TOR activation was essential for the lifespan-promoting effect of flies against natural aging-induced oxidative stress. For example, rice protein hydrolysates increased flies’ longevity by boosting the gene expression in Nrf2 and TOR signaling pathways [14]. Peptides derived from crimson snapper scales extended the lifespan of flies and harmful environmental exposure triggered oxidative stress by inhibiting the TOR activation [13]. Moreover, it has been demonstrated that rapamycin, a TORC1 inhibitor, activates SKN-1 (the ortholog of mammalian Nrf2) to prolong the longevity of nematodes [47]. STP significantly downregulated the *TORC* expression, suggesting that STP regulated the TOR signaling pathway to inhibit oxidative stress in flies. Additionally, inhibition of TORC1 could regulate the process of autophagy, which also has a central role in promoting longevity [15]. During the process of autophagy, lysosomes will degrade the damaged proteins, lipids, nucleic acids, and sugars, which gives cell energy and eliminates damaged cell components to exert a protective function for the cell [48]. The lifespan of flies was extended by over-expressing the autophagy kinase *Atg1* and *Atg8* [49,50]. As expected, the expression of the *Atg1* and *Atg8a* were upregulated in the current study, implying that STP promoted autophagy. During starvation, autophagy in animals mainly progresses for optimal survival [49]. STP improved the flies’ tolerance to starvation stress and supported enhanced autophagy. In addition, oxidative stress and autophagy are linked via the TORC1 [15]. Namely, STP may inhibit the TOR signaling pathway to prolong the lifespan of flies. Similarly, walnut-derived peptides protected PC12 cells from oxidative stress by promoting autophagy through the Akt/TOR signaling pathway [9].

Rapamycin, an inhibitor of TORC1, could bind to the domain of FKBP-rapamycin binding (FRB) of TOR by forming a complex with the 12-kDa FK506-binding protein FKBP12, thereby inhibiting the physiological activity of TOR [15]. Therefore, the identified peptide sequences from STP were combined with FKBP12-FRB by molecular docking, and their interactions were analyzed; the responsible potential anti-aging peptides in STP were further determined in this study. As a result, 95.18% of peptides from the identified sequences in STP could successfully dock with FKBP12-FRB. The representative peptide sequences ADLETYLLEKSRVT could form a stable ternary complex with FKBP12-FRB through hydrogen bonds, van der Walls, hydrophobic interactions, and electrostatic interactions. Therefore, the peptide sequences in STP could form a stable ternary complex with FKBP12-FRB, thereby inhibiting the expression of the *TOR* gene from exerting anti-aging activity.

## 5. Conclusions

In this study, the supplementation of diets with STP effectively extended lifespan, improved healthspan, and enhanced stress resistances of oxidation, heat, and starvation in flies. Furthermore, STP decreased oxidative stress and enhanced autophagy by downregulating the expression of TOR, thus prolonging the lifespan of flies. Based on the molecular docking results, 95.18% of peptides with a potential inhibitory effect on TOR were identified from STP, and ADLETYLLEKSRVT could form a stable ternary complex with FKBP12-FRB by hydrogen bonds, van der Walls, hydrophobic interactions, and electrostatic interactions. Overall, this study has provided direct evidence of a longevity-promoting effect of soft-shell turtle peptides and revealed it could serve as a healthy supplementation in enhancing lifespan and healthspan. Importantly, the relationship between STP and gut microbiota in flies will be investigated in future studies.

## Data Availability

Data are contained within the article.

## Supplementary Materials

The following supporting information can be downloaded at: https://www.mdpi.com/article/10.3390/nu14245205/s1. Table S1: Sequences of primers, Table S2: Peptide sequences identified from STP and their docking energy to FKBP12-FRB.
