# Supplementary material for: Soft-Shelled Turtle Peptides Extend Lifespan and Healthspan in Drosophila
Source: Nutrients. 2022 Dec 7;14(24):5205. doi: 10.3390/nu14245205 (PMC9781693; doi:10.3390/nu14245205)
Supplement: Supplementary file 1 [file nutrients-14-05205-s001.zip › nutrients-2021333-supplementary.pdf]

Supplementary Files

Table S1 Sequences of primers used in this study.

Gene name	Forward primer (5'-3')	Reverse primer (5'-3')
Rp49	GACAGTATCTGATGCCCAACA	CTTCTTGGAGGAGACGCCGT
Hsp70	ACCAAGGGGTGTGCCCCAGA	CTTGGCCTTGCCCGTGCTCA
Nrf2	GTCGCCACTAAAACCGCATC	TTGTTCTTTCCACGCCGACG
Keap1	GCGCTCGTCAGCCCATTTT	GGATGCGCATAATTCCTCTTCTT
Ho-1	ATGACGAGGAGCAGCAGAAG	ACAAAGATTAGTGCGAGGGC
TORC	GGCCGTCCAGGTTCAAAAAC	ATGGACAACCGCTTTAGCCA
Atg1	GCTTCTTTGTTCACCGCTTC	GCTTGACCAGCTTCAGTTCC
Atg8a	AGTCCCAAAAGCAAACGAAG	TTGTCCAAATCACCGATGC

Table S2 Peptide sequences identified from STP and their docking energy to FKBP12-FRB.

No.	Sequence	ALC (%)	Mass (Da)	(-) CDOCKER Energy (kcal/mol)
#1	A(+42.01)GPR	98.3	441.2336	64.4704
#2	LDFPR	98.1	646.3438	91.3283
#3	PPHL	98	462.2591	54.1084
#4	A(+42.01)MTN(+0.98)LLSAADLK	97.8	1289.6537	F
#5	A(+42.01)GLPR	97.8	554.3176	77.4159
#6	LKPLL	97.7	582.4105	94.0279
#7	HRGF	97.7	515.2604	90.5414
#8	PAN(+0.98)LK	97.6	542.3064	86.1805
#9	M(+42.01)DALK	97.3	618.3047	82.475
#10	A(+42.01)GER	97.3	473.2234	93.5502
#11	A(+42.01)GLPH	97.2	535.2755	82.5445
#12	M(+42.01)DAL	97.1	490.2097	86.4313
#13	LLKL	97.1	485.3577	97.587
#14	APPHL	97	533.2961	64.5419
#15	LAGPK	96.9	484.3009	86.6101
#16	HWPW	96.9	624.2808	76.1215
#17	FERVL	96.9	662.3751	113.041
#18	LN(+0.98)FPK	96.7	618.3377	93.4555
#19	LGPR	96.7	441.27	72.5111
#20	YMLPVH	96.6	758.3785	106.356
#21	K(+42.01)LDYKDLVHL	96.6	1284.7078	168.737
#22	FN(+0.98)LH	96.5	530.2489	95.7122
#23	LSGPR	96.4	528.302	83.2967
#24	FKDL	96.4	521.2849	103.944
#25	DLDLR	96.3	630.3337	122.497
#26	Q(+42.01)LPH	96.1	535.2755	74.57
#27	LPHLS	96.1	565.3224	92.5178
#28	LKDPF	96	618.3377	103.49
#29	K(+42.01)LTLK	95.8	643.4268	122.288

#30	PVLPH	95.7	561.3275	69.6856
#31	LLAPPE	95.7	638.3639	83.3049
#32	YNVR	95.5	550.2863	96.4848
#33	SN(+0.98)LAH	95.5	541.2496	107.033
#34	LAGPR	95.5	512.3071	80.4295
#35	ETLLPR	95.5	727.4228	107.417
#36	C(+42.01)LFN(+0.98)HHM	95.5	943.368	119.17
#37	WLPR	95.4	570.3278	78.006
#38	TPPFEV	95.4	688.3431	92.064
#39	RDLK	95.3	530.3176	107.534
#40	WKPPL	95.2	639.3744	76.9192
#41	TEKEL	95.2	618.3224	138.182
#42	LRLF	95.1	547.3482	91.6427
#43	WRPPQP	95	779.4078	55.3785
#44	LELPR	95	626.3751	96.6594
#45	FAHL	94.9	486.2591	90.7303
#46	FHLL	94.7	528.306	92.4053
#47	EAPPHL	94.7	662.3387	95.7631
#48	WGDAGAE	94.6	704.2766	131.176
#49	LPGEF	94.6	561.2798	94.1741
#50	HGLT	94.5	426.2227	84.9218
#51	FKLL	94.5	519.342	96.7764
#52	AVKVL	94.5	528.3635	114.909
#53	WEPPR	94.4	683.3391	82.8374
#54	LPHL	94.4	478.2903	72.4017
#55	PAWE	94.3	501.2223	68.7767
#56	FRLV	94.3	533.3325	91.5826
#57	AAGPA	94.3	385.1961	66.9739
#58	YDLDF	94.2	671.2802	122.118
#59	SFLPH	94.2	599.3067	89.8858
#60	TFDE	94.1	510.1962	94.8037
#61	KEEL	94.1	517.2748	119.858
#62	Q(+42.01)LPR	94	554.3176	75.2764
#63	LELLPR	94	739.4592	91.9676
#64	WDMPR	93.9	703.3112	95.9526
#65	STGVFTTTEKASAHL	93.9	1548.7783	218.342
#66	GPRL	93.8	441.27	67.8012
#67	FQLK	93.8	534.3166	100.645
#68	C(+42.01)LFDHHM	93.8	943.368	117.078
#69	AVFPR	93.8	588.3384	82.8597
#70	WRPPGAP	93.7	779.4078	73.3435
#71	TPGAM	93.7	475.2101	73.7155
#72	YFAH	93.6	536.2383	96.7154
#73	PVLPHQ	93.6	689.386	87.6311

#74	HLEF	93.6	544.2645	96.59
#75	FRYL	93.6	597.3275	96.2403
#76	VVLHL	93.5	579.3744	107.553
#77	PFRP	93.4	515.2856	60.2315
#78	LPGEL	93.4	527.2955	86.2818
#79	LLLK	93.4	485.3577	95.2735
#80	YRLL	93.3	563.3431	96.2721
#81	NLLPR	93.3	611.3755	88.5929
#82	LLLPR	93.3	610.4166	80.6791
#83	FKPF	93.3	537.2951	83.5065
#84	LGER	93.2	473.2598	92.4909
#85	FPHFDLHHDSEQV	93.2	1606.7164	F
#86	DLLHDPL	93.2	821.4283	114.295
#87	FGPHFDLHHDSEQ	93.1	1564.6694	170.756
#88	LDRPF	93	646.3438	92.4031
#89	FGEK	93	479.238	107.038
#90	EAFSLFDRTPK	93	1309.6666	F
#91	ADLETYLLEKSRVT	93	1636.8672	225.071
#92	LPHSL	92.9	565.3224	83.2045
#93	LPDSV	92.9	529.2748	88.9039
#94	ELWDW	92.9	747.3228	111.061
#95	APSGGF	92.9	534.2438	82.6981
#96	K(+42.01)WAKVESDLPAH	92.8	1421.7302	173.678
#97	DWRPPQP	92.8	894.4348	89.4745
#98	QAGPA	92.7	442.2176	78.3234
#99	LKLL	92.7	485.3577	95.3038
#100	FRVL	92.7	533.3325	89.0986
#101	APSGGFDF	92.7	796.3391	110.318
#102	TN(+0.98)LK	92.6	475.2642	93.3657
#103	SLGS	92.6	362.1801	71.6459
#104	LVEK	92.6	487.3006	107.487
#105	K(+42.01)WAKVESDLPAHGQ	92.6	1606.8103	F
#106	GLHE	92.6	454.2176	103.32
#107	FKVF	92.6	539.3107	97.1179
#108	LWAKDL	92.5	744.417	124.838
#109	AAGPL	92.4	427.2431	69.2422
#110	AAGPAGN	92.4	556.2605	F
#111	A(+42.01)GPQ	92.4	413.191	62.8493
#112	YFPH	92.3	562.254	85.9691
#113	WKPPV	92.3	625.3588	83.1348
#114	VVDGV	92.3	487.2642	101.887
#115	TFYDEL	92.3	786.3436	126.189
#116	PPLPR	92.3	578.354	51.6889
#117	LPEL	92.3	470.274	76.2688

#118	HN(+0.98)VPL	92.3	579.3016	82.7674
#119	HELE	92.3	526.2387	108.591
#120	AAPFL	92.3	517.29	108.591
#121	LPVGPF	92.2	628.3584	76.43
#122	LDLPR	92.2	612.3595	94.9403
#123	GLHET	92.2	555.2653	116.814
#124	SGGFDF	92.1	628.2493	109.523
#125	PPKE	92.1	469.2536	66.9285
#126	PLSLPH	92.1	662.3751	75.0519
#127	MFGPA	92.1	521.2308	76.4036
#128	FPHFDLHHDSEQ	92.1	1507.6479	F
#129	R(+42.01)YFH	92	663.3129	103.201
#130	K(+42.01)LTDW	92	703.3541	123.868
#131	AAPF	92	404.206	60.6935
#132	YHLF	91.9	578.2853	93.7385
#133	QLGPA	91.9	484.2645	76.9177
#134	HLFY	91.9	578.2853	90.3434
#135	FLLK	91.9	519.342	101.281
#136	TGEF	91.8	452.1907	85.0555
#137	LGGL	91.7	358.2216	73.117
#138	EKHATEDKVKDLTEEM	91.7	1901.9041	220.22
#139	SSYL	91.6	468.222	85.4523
#140	KLPF	91.6	503.3108	76.1469
#141	A(+42.01)PWPR	91.6	667.3442	67.3409
#142	T(+42.01)ALPPW	91.5	725.3748	82.5971
#143	PGER	91.5	457.2285	70.6997
#144	LEVPH	91.5	593.3173	70.6997
#145	LALL	91.5	428.2998	81.501
#146	ELEE	91.5	518.2224	113.132
#147	KDFELS	91.4	737.3596	147.905
#148	FGPHFDLHHDSEQV	91.3	1663.7379	219.525
#149	A(+42.01)PVN(+0.98)SAYEVLKLK	91.3	1473.8079	F
#150	LPPLPK	91.2	663.4319	74.7236
#151	FPLK	91.2	503.3108	78.0927
#152	A(+42.01)LGPW	91.2	584.2958	76.5635
#153	YLN(+0.98)LPK	91.1	747.4167	113.626
#154	AAGL	91.1	330.1903	73.1339
#155	WERPF	91	733.3547	106.347
#156	SLN(+0.98)R	91	489.2547	87.0465
#157	K(+42.01)FPS	90.9	519.2693	74.071
#158	TGLVGP	90.7	542.3064	90.1345
#159	LPLL	90.7	454.3155	67.3547
#160	LPGDW	90.7	586.2751	87.3964
#161	WNLR	90.6	587.318	87.3964

#162	TVDT	90.6	434.2013	89.8831
#163	TEAPL	90.6	529.2748	90.3714
#164	FYDEL	90.6	685.2959	127.324
#165	NFDF	90.5	541.2172	71.6382
#166	EFGL	90.5	464.2271	92.6906
#167	WEPPVKP	90.4	851.4541	91.4445
#168	PVVP	90.4	410.2529	40.8515
#169	M(+42.01)N(+0.98)ALKKKMQM	90.4	1264.6342	F
#170	LALA	90.4	386.2529	74.7811
#171	WGPY	90.3	521.2274	69.7525
#172	M(+42.01)DALLK	90.3	731.3887	125.406
#173	FSLPH	90.3	599.3067	61.8252
#174	ALGPA	90.3	427.2431	73.7527
#175	TLDLH	90.2	597.3122	116.571
#176	SVGGL	90.2	431.238	87.3185
#177	LSN(+0.98)LSDLAH	90.2	969.4767	124.46
#178	VPGLTF	90.1	632.3533	93.8618
#179	TPGA	90.1	344.1696	54.2774
#180	K(+42.01)LN(+0.98)YRLSVH	90.1	1171.6349	F
#181	DLLR	90.1	515.3067	99.1765
#182	APSGGFD	90.1	649.2708	99.5067
#183	WGLPR	90	627.3492	85.3147
#184	WDPW	90	602.2489	77.6375
#185	VPVV	90	412.2686	62.5616
#186	QTGPS	90	488.2231	84.7256
#187	FKDW	90	594.2802	105.426
